# Oleoylethanolamide Protects against Acute Ischemic Stroke by Promoting PPARα-Mediated Microglia/Macrophage M2 Polarization

**DOI:** 10.3390/ph16040621

**Published:** 2023-04-20

**Authors:** Ying Li, Yanan Zhang, Qing Wang, Chuang Wu, Guicheng Du, Lichao Yang

**Affiliations:** 1 Department of Pharmacy, Xiamen Medical College, Xiamen 361023, China; 2 Xiamen Key Laboratory of Traditional Chinese Medicine Bioengineering, Xiamen Medical College, Xiamen 361023, China; 3 School of Medicine, Xiamen University, Xiamen 361005, China

**Keywords:** oleoylethanolamide, PPARα, microglia, polarization, ischemic stroke

## Abstract

Oleoylethanolamide (OEA) has been demonstrated to be a feasible protectant in ischemic stroke. However, the mechanism for OEA-afforded neuroprotection remains elusive. The present study aimed to investigate the neuroprotective effects of OEA on peroxisome proliferator-activated receptor α (PPARα)-mediated microglia M2 polarization after cerebral ischemia. Transient middle cerebral artery occlusion (tMCAO) was induced for 1 h in wild-type (WT) or PPARα-knock-out (KO) mice. Mouse small glioma cells (BV2) microglia and primary microglia cultures were used to evaluate the direct effect of OEA on microglia. A coculture system was used to further elucidate the effect of OEA on microglial polarization and ischemic neurons’ fate. OEA promoted the microglia switch from an inflammatory M1 phenotype to the protective M2 phenotype and enhanced the binding of PPARα with the arginase1 (Arg1) and Ym1 promoter in WT mice but not in KO mice after MCAO. Notably, the increased M2 microglia caused by OEA treatment were strongly linked to neuron survival after ischemic stroke. In vitro studies confirmed that OEA shifted BV2 microglia from (lipopolysaccharide) LPS-induced M1-like to M2-like phenotype through PPARα. Additionally, the activation of PPARα in primary microglia by OEA led to an M2 protective phenotype that enhanced neuronal survival against oxygen-glucose deprivation (OGD) in the coculture systems. Our findings demonstrate the novel effects of OEA in enhancing microglia M2 polarization to protect neighboring neurons by activating the PPARα signal, which is a new mechanism of OEA against cerebral ischemic injury. Therefore, OEA might be a promising therapeutic drug for stroke and targeting PPARα-mediated M2 microglia may represent a new strategy to treat ischemic stroke.

## 1. Introduction

Ischemic stroke is a severe CNS disease, and limited effective therapies available for the treatment of ischemic stroke. Recombinant tissue plasminogen activator is the only clinical treatment approved by the United States Food and Drug Administration for stroke. However, the short half-time and extremely narrow therapeutic time window restrict its clinic application [1]. Although several promising drugs have been identified in preclinical studies, no neuroprotective molecule has been reported to show clinical efficacy in human trials [2,3]. Thus, finding new effective drugs or strategies for treatment post-stroke is imperative. Previous reports have described the neuroprotective effects of oleoylethanolamide (OEA) on animal models of different neurological disorders [4]. Moreover, our previous studies showed that OEA significantly ameliorated the infarct volume, attenuated neuronal apoptosis on acute cerebral ischemia and markedly improved spatial cognitive deficits at the later stages of ischemic stroke [5,6,7]. Furthermore, we also indicated that the integration of a phospholipid-complex nanocarrier assembly with OEA was an efficient stroke therapy by dramatically inhibiting the inflammatory response and improving both motor and cognitive functions [8]. Thus, as a promising neuroprotective drug, the therapeutic effects and mechanisms in ischemic stroke of OEA are worthy of further assessment.

Cerebral ischemia leads to multiple rapid immune responses, such as the activation of resident microglia, the infiltration of peripheral macrophages and the accumulation of immune mediators in the injured area [9]. An increasing number of studies now agree that activated microglia/macrophages assume distinct phenotypes and engage diverse functions in the injured brain [10]. For instance, classically activated M1 microglia may exacerbate brain injury by releasing neurotoxic substances. In contrast, alternatively activated M2 microglia possess neuroprotective properties by removing cell debris and releasing trophic factors [10]. Microglia/macrophage phenotype changes dynamically from M2 to M1 during chronic inflammation after a stroke [11]. This M2-to-M1 shift after stroke expands neuronal damage and reduces their self-restorative abilities. Therefore, searching for drugs that modulate microglia/macrophages toward the beneficial M2 phenotype may be a new anti-inflammatory strategy to protect against ischemic stroke. OEA is a potent endogenous ligand of peroxisome proliferator-activated receptor α (PPARα) [12]. PPARα was demonstrated to participate in the regulation of blood lipids [12], inhibition of inflammation [13] and promotion of learning and memory [14]. Additionally, our study showed that PPARα signaling is involved in the inhibition of glial scarring after stroke [15]. However, whether PPARα participates in the microglia M1-M2 phenotype switch in inflammation after stroke has not yet been explored. Although the neuroprotective effect of OEA in ischemic stroke has been confirmed in our previous reports, the exact neuroprotective mechanisms of OEA are still unknown, especially in regard to the microglial M1/M2 polarization in cerebral ischemic injury. 

In the present study, the ability of OEA to regulate M2 microglial polarization was investigated both in vivo and in vitro. Additionally, the effects of OEA on M2 microglia-mediated neuroprotection were also explored. Furthermore, we examined whether the regulation of M2 microglia polarization and neuroprotection of OEA is related to microglial PPARα signaling. These data provide evidence that OEA exerts a neuroprotective effect in ischemic stroke by shifting microglia polarization from M1 to M2 through PPARα signaling.

## 2. Results

### 2.1. OEA Attenuates Microglia/Macrophage M1 Polarization in Middle Cerebral Artery Occlusion (MCAO) Mice through the PPARα Pathway

**Figure 1:**
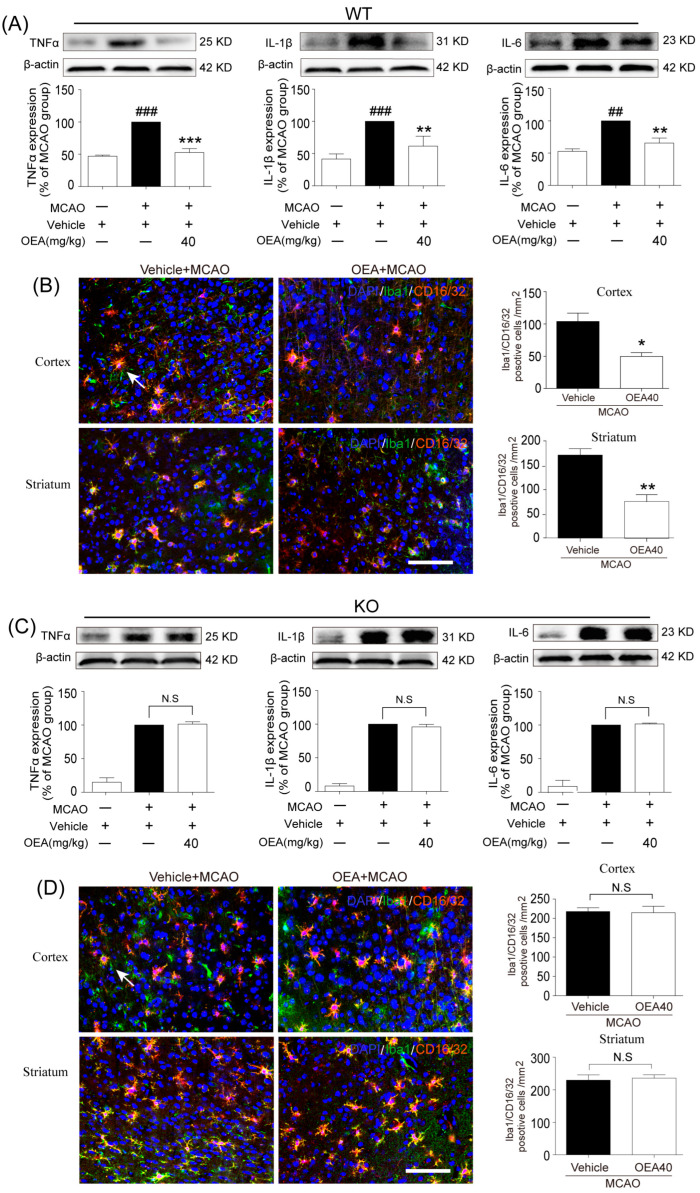
Oleoylethanolamide (OEA) treatment suppresses microglia M1 polarization in the injured cortex and striatum in wild-type (WT) but not PPARα-knock-out (KO) mice at 3 days after middle cerebral artery occlusion (MCAO). (**A**) Representative western blot images and quantitative analysis of the expression levels of TNF-α, IL-1β and IL-6 in injured brain tissue of WT mice on day 3 after MCAO. (**B**) Representative immunofluorescence images and quantification of CD16/32 and Iba-1 in the peri-infarction of WT mice on day 3 after MCAO (Iba1^+^/CD16/32^+^ cells were shown by white arrow). (**C**) Representative western blot images and quantitative analysis of the expression levels of TNF-α, IL-1β and IL-6 in injured brain tissue of KO mice on day 3 after MCAO. (**D**) Representative immunofluorescence images and quantification of CD16/32 and Iba-1 in the peri-infarction of KO mice on day 3 after MCAO (Iba1^+^/CD16/32^+^ cells were shown by white arrow). The data are the means ± SEM. *n* = 5 per group. ^##^ *p* < 0.01, ^###^ *p* < 0.001 vs. vehicle + sham; * *p* < 0.05, ** *p* < 0.01, *** *p* < 0.001 vs. MCAO + vehicle group; N.S = No Significance. Scale Bar = 100 μm.

As microglia/macrophage polarization is dynamic following the pathological progression of ischemic stroke [16], we chose one time point (3 d after the cerebral ischemic injury) to detect whether OEA was able to affect the microglia/macrophage polarization at the penumbra field. First, we explored the effect of OEA on microglia/macrophage M1 polarization after MCAO. Western blot analysis showed that the expression of M1 markers (tumor necrosis factor (TNF)-α, interleukin (IL)-1β and IL-6) in the injured brain tissue of wild-type (WT; Sv129) mice were all increased after MCAO. However, OEA reversed the trend of M1 markers (Figure 1A). Other central nervous cells or infiltrating immune cells also express M1 and M2 signature proteins. Therefore, the western blot data on the injured brain tissue reflect a mixture of cell types. To specifically assess the polarization state of microglia/macrophages after stroke, representative M1-like (CD16/32) or M2-like (CD206) marker proteins were analyzed by double immunofluorescent staining with the microglia/macrophage marker Iba1 in the penumbral region of cortex and the striatum of mice after MCAO. Consistent with the western blot results, the M1 marker CD16/32 was highly expressed in Iba1^+^ cells (microglia/macrophage) in the ischemic penumbra of the cortex and striatum, but OEA treatment obviously decreased the Iba1^+^/CD16^+^/32^+^ cell number after MCAO (Figure 1B). To examine whether the inhibitory effect of OEA on microglia/macrophage M1 polarization was specifically mediated by PPARα signaling, we further performed focal cerebral ischemia in PPARα-knock-out (KO) mice. Interestingly, we found no differences in the expression of M1 markers between the vehicle + MCAO group and the OEA+MCAO group in PPARα-KO mice after MCAO (Figure 1C,D). These results indicated that OEA attenuated microglia/macrophage M1 polarization after cerebral ischemia via the PPARα pathway.

### 2.2. OEA Shifts Microglia/Macrophage Polarization toward the M2 Phenotype through PPARα Signaling

**Figure 2:**
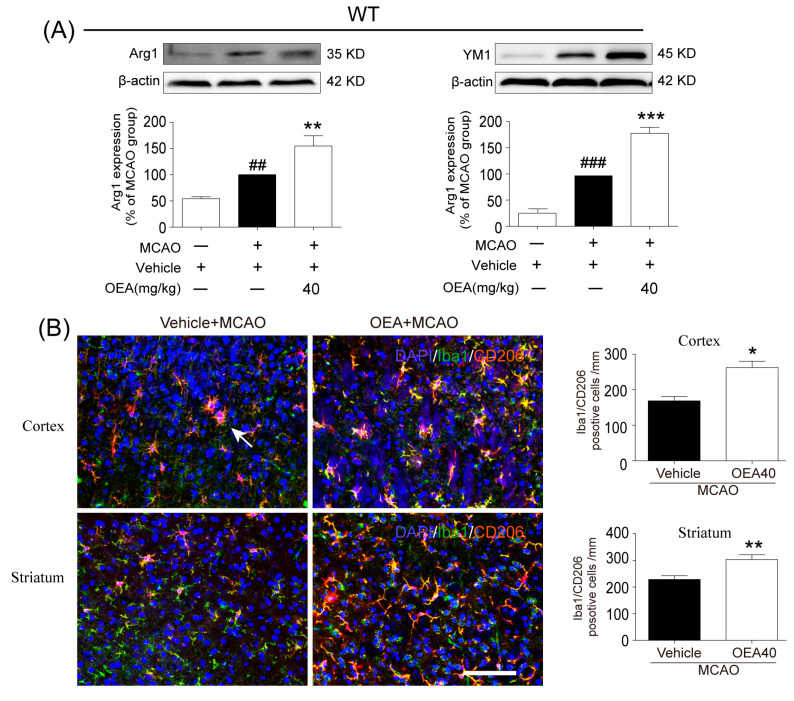

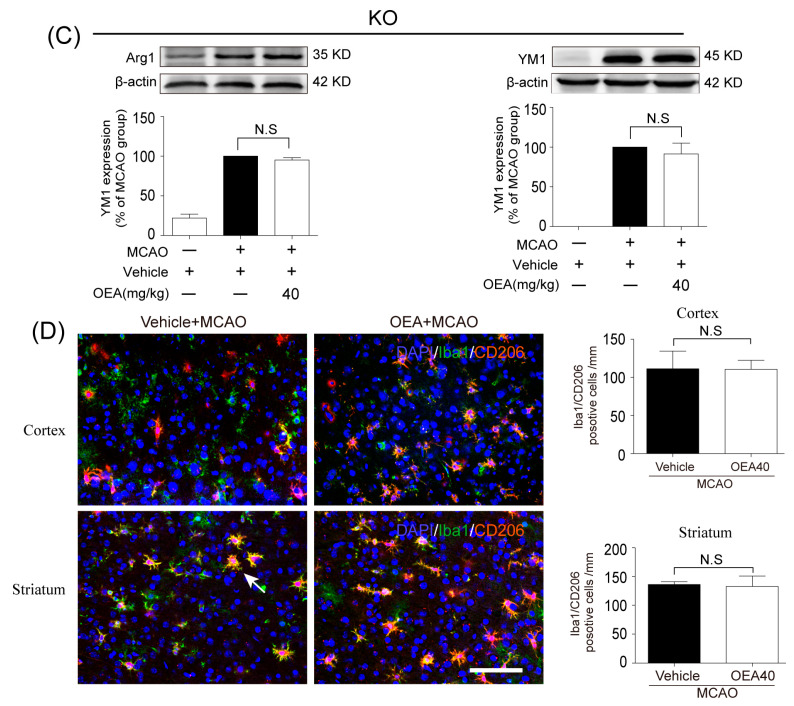
OEA treatment enhances microglia M2 polarization in the injured cortex and striatum in WT but not KO mice at 3 days after MCAO. (**A**) Representative western blot images and quantitative analysis of the expression levels of Arg1 and YM1 in injured brain tissue of WT mice on day 3 after MCAO. (**B**) Representative immunofluorescence images and quantification of CD206 and Iba-1 in the peri-infarction of WT mice on day 3 after MCAO (Iba1^+^/CD206^+^ cells were shown by white arrow). (**C**) Representative western blot images and quantitative analysis of the expression levels of Arg1 and YM1 in injured brain tissue of KO mice on day 3 after MCAO. (**D**) Representative immunofluorescence images and quantification of CD206 and Iba-1 in the peri-infarction of KO mice on day 3 after MCAO (Iba1^+^/CD206^+^ cells were shown by white arrow). The data are the means ± SEM. *n* = 5 per group. ^##^ *p* < 0.01, ^###^ *p* < 0.001 vs. vehicle + sham; * *p* < 0.05, ** *p* < 0.01, *** *p* < 0.001 vs. MCAO + vehicle group; N.S = No Significance. Scale Bar = 100 μm.

Next, we investigated whether OEA promotes the polarization of microglia/macrophages toward the M2 phenotype after MCAO. M2 markers (Arg1, YM1 and CD206) were examined to analyze the microglia/macrophage phenotype in the penumbra field of WT and PPARα-KO mice after MCAO. We found that the expression of markers Arg1 and YM-1 was increased after MCAO. Interestingly, OEA further promoted the levels of the M2 markers (Figure 2A). Meanwhile, the co-expression of the M2 marker CD206 and Iba1 was higher in the MCAO+OEA group compared with the MCAO + vehicle group (Figure 2B), suggesting that OEA promotes the migration or infiltration of M2-like microglia/macrophages into the infarct border after cerebral ischemia. To explore whether OEA influenced the microglia/macrophage M2 phenotypes through the PPARα pathway, we next examined the expression of the M2 markers Arg1, YM1 and CD206 in KO mice after MCAO. However, we did not detect differences in M2 marker expression between the vehicle + MCAO and OEA+MCAO groups in PPARα-KO mice (Figure 2C,D). These results indicated that OEA promotes the polarization of microglia/macrophages toward the M2 phenotype after cerebral injury in a PPARα-dependent-manner.

We further used the microglia-specific marker TMEM119 and M1-like (iNOS, CD16/32) or M2-like (YM1, CD206) marker proteins to analyze the effect of OEA on microglia polarization by immunofluorescent staining in the penumbral region of cortex and the striatum of mice after MCAO. We found that OEA inhibited the TMEM119^+^/iNOS^+^/CD16/32^+^ expression and promoted TMEM119^+^/Ym1^+^/CD206^+^ expression in WT mice but not in PPARα KO mice. These results further demonstrate OEA shifted microglia from M1-like to M2-like phenotype through the PPARα pathway (Supplementary Figures S1 and S2).

### 2.3. OEA-Induced Microglia/Macrophage M2 Phenotype Correlates with Neuroprotection after Ischemic Stroke

**Figure 3:**
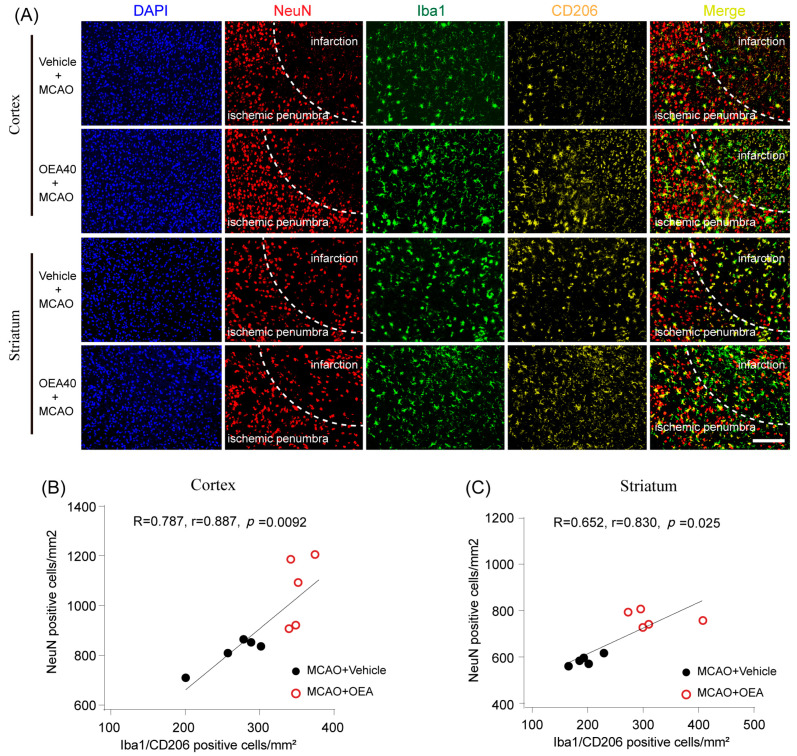
Microglia M2 phenotype elicited by OEA is positively linked with neuron survival after stroke. (**A**) Representative images of NeuN, Iba1 and CD206 triple-staining at 3 d after ischemic stroke. (**B**,**C**) There was a positive correlation between the number of cells positive for NeuN and Iba1/CD206 positive cells in the injured cortex and striatum at 3 d after MCAO. The data are the means ± SEM. *n* = 5 per group. Scale Bar = 100 μm.

To further investigate whether the neuroprotective effects of OEA correlate with the microglia/macrophage M2 phenotype, brains were subjected to immunostaining for NeuN, Iba1 and CD206. Then, we performed Pearson product linear regression analysis to assess the correlation between the NeuN positive cells and M2 microglia/macrophages. Interestingly, the number of NeuN-stained cells positively correlated with the number of Iba1/CD206 positive cells in the penumbra of the cortex and striatum after MCAO (Figure 3A–C). These results indicate that the microglia/macrophage phenotypic switch from M1 to M2 elicited by OEA treatments may contribute to enhancing neuron survival after stroke.

### 2.4. Arg1 and Ym1 *Are Directly Regulated by OEA-Mediated PPARα Activation*

**Figure 4:**
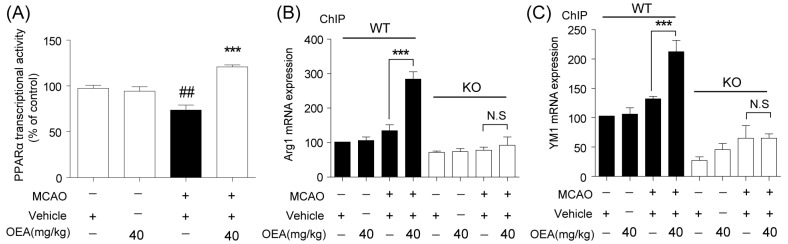
The activation of PPARα by OEA transcriptionally modulates Arg1 and Ym1 expression in WT mice cortex after MCAO. (**A**) The activation of PPARα was analyzed by EMSA at 24 h after MCAO. (**B**,**C**) The binding activity of PPARα with the Arg1 and YM1 promoters after MCAO was detected using a ChIP assay with a PPARα antibody at 24 after MCAO. The data are the means ± SEM. *n* = 5 per group. ^##^ *p* < 0.01 vs. vehicle + sham group, *** *p* < 0.001 vs. vehicle + MCAO group; N.S = No Significance.

It has been reported that PPARα is involved in neuroprotection in ischemic stroke. To detect whether OEA enhances the expression of M2 markers by promoting PPARα transcriptional activity, an electrophoretic mobility shift assay (EMSA) was performed 24 h after MCAO. The data showed that the PPARα-DNA binding activity was significantly decreased in the vehicle + MCAO group compared to the vehicle + sham group, but this change was reversed by OEA treatment (Figure 4A). These results indicate OEA increases the activation of PPARα in injured brain tissue after cerebral ischemia. 

Next, we determined whether the M2 genes Arg1 and YM1 are the direct PPARα target genes and if OEA enhances microglia/macrophage M2 polarization through PPARα signaling. ChIP assays were performed using primers for the PPARα binding sites in the Arg1 and YM-1 promoters. We found that the binding activity of PPARα with the Arg1 and YM1 promoters was increased in the OEA+MCAO group compared to the vehicle + MCAO group at 24 h after MCAO in WT mice (Figure 4B,C), but these changes were not observed in PPARα-KO mice (Figure 4B,C). These results confirmed that OEA promotes microglia/macrophage M2 polarization after MCAO through the PPARα pathway.

### 2.5. OEA Inhibits Microglia M1 Polarization and Facilitates Microglia M2 Polarization In Vitro

**Figure 5:**
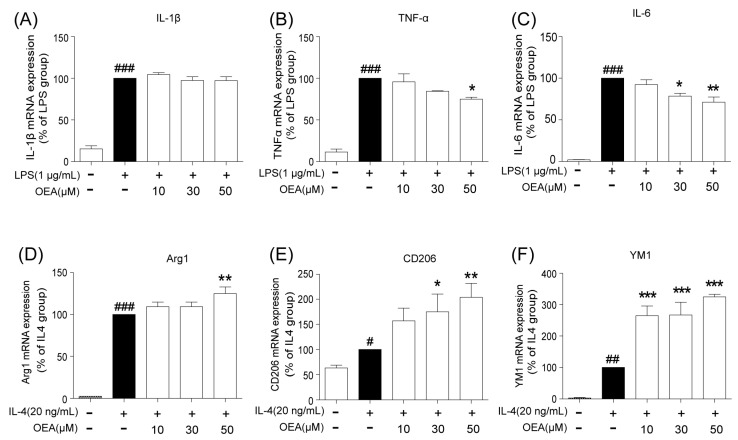
OEA inhibits LPS-induced M1 polarization and further promotes IL-4-induced M2 polarization in BV2 microglial cells. BV2 microglia were stimulated with LPS (1 ug/mL) or L4 (20 ng/mL) and then treated with vehicle or 10, 30 and 50 μM OEA. (**A**–**C**) The mRNA expression of M1 markers was examined by RT-PCR. (**D**–**F**) The mRNA expression of M2 markers was examined by RT-PCR. Samples were collected from five independent experiments, each performed in duplicate. The data are the means ± SEM. *n* = 5 per group. ^#^ *p* < 0.05, ^##^ *p* < 0.01, ^###^ *p* < 0.001 vs. the control (CON) group; * *p* < 0.05, ** *p* < 0.01, *** *p* < 0.001 vs. LPS-induced or IL-4-inuced group.

First, we performed an MTT assay to examine OEA cytotoxicity in mouse small glioma (BV2) cells. Treatment with 100 μM OEA showed a significant cytotoxic effect on BV2 microglia cells. OEA at dosages of 10, 30 and 50 μM exerted no significant effects on microglial survival (Supplementary Figure S3). Therefore, the concentrations 10, 30 and 50 μM were selected for further experiments. BV2 cells were treated with (lipopolysaccharide) LPS (1 μg/mL) for 12 h to induce M1 phenotype, and IL-4 (20 ng/mL) was used for M2 induction for the same period. We then investigated the different concentrations of OEA (10, 30 and 50 μM) on BV2 polarization using qRT-PCR to detect the M1 biomarker and M2 biomarker. The data showed that the mRNA expression of IL-1β (M1 marker) was drastically upregulated by LPS (Figure 5A) and not significantly reduced with OEA treatment (Figure 5A). However, the mRNA expression of M1 markers (TNF-α and IL-6) was markedly increased in LPS-induced BV2 microglia (Figure 5B,C) but significantly reduced after treatment with 30 or 50 μM OEA (Figure 5B,C). Additionally, OEA further promoted the IL-4-induced upregulation of Arg-1, CD206 and YM-1, the M2 markers, in BV2 cells in a dose-dependent manner (Figure 5D–F). Therefore, our data demonstrate that OEA could directly modulate microglia polarization.

### 2.6. OEA Shifts LPS-Induced Microglia from the M1 Phenotype to the M2 Phenotype through the PPARα Pathway

**Figure 6:**
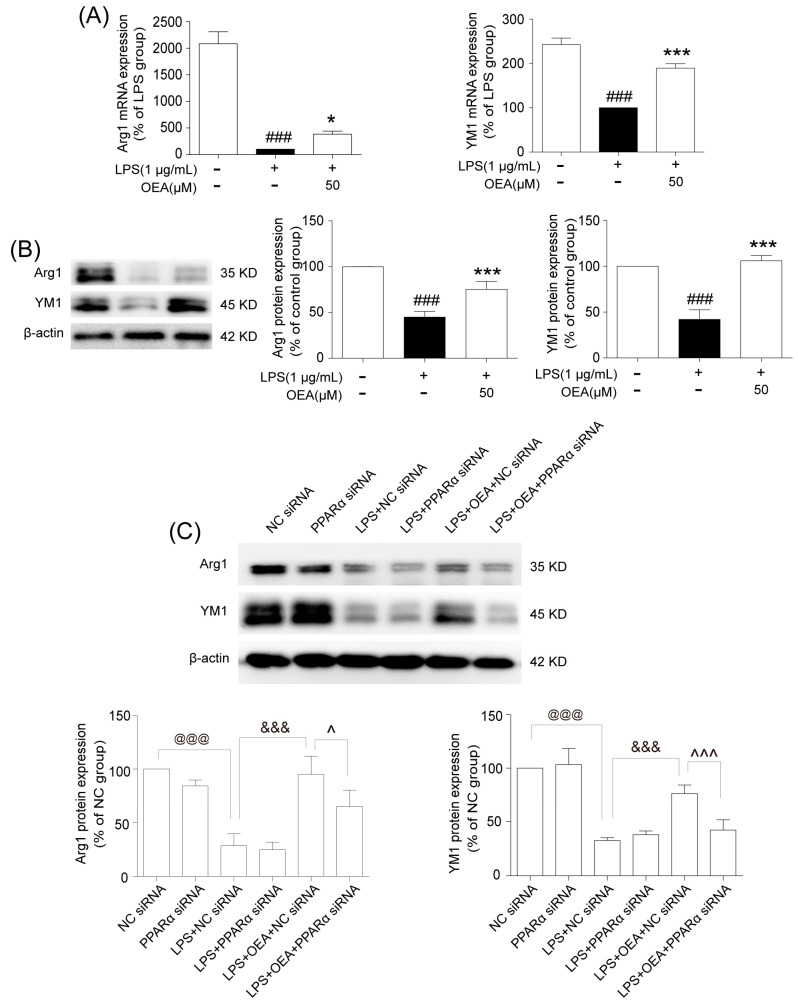
OEA switched microglia from an M1-like phenotype to an M2-like phenotype through a PPARα-dependent pathway. (**A**) The effect of OEA on Arg1 and YM1 mRNA expression in LPS-induced BV2 cells. (**B**) The effect of OEA on Arg1 and YM1 protein expression in LPS-induced BV2 cells. (**C**) The knock-down of PPARα antagonized the effect of OEA on the microglial M1-M2 shift in LPS-induced BV2 cells. The results are presented as percentages compared with the NC group, LPS group or the NC siRNA group (set to 100%). Samples were collected from five independent experiments, each performed in duplicate. The data are the means ± SEM. *n* = 5 per group. ^###^ *p* < 0.001 vs. Control group, * *p* < 0.05, *** *p* < 0.001 vs. LPS group; ^@@@^ *p* < 0.001 vs. NC siRNA; ^&&&^ *p* < 0.001 vs. LPS+NC siRNA; ^^^ *p* < 0.05, ^^^^^ *p* < 0.001 vs. LPS+OEA+NC siRNA.

To further test whether OEA can shift microglia from an M1-like to M2-like phenotype, the BV2 cells were pretreated with OEA at 50 μM or 0.04% DMSO for 2 h before exposure to LPS (1 μg/mL) for an additional 12 h or 24 h. The stimulation of BV2 cells with LPS markedly decreased the mRNA and protein expression of Arg-1 and YM-1 (Figure 6A,B). However, these changes were reversed with OEA treatment (Figure 6A,B). These results indicated that OEA promotes the transformation of microglia phenotypically from an M1-like to an M2-like phenotype.

Next, we further identified the mechanism that controls phenotypic switching in microglia. BV2 cells were transiently transfected with PPARα siRNA for 24 h, and the knock-down efficiencies of PPARα were 67.8%, as determined by western blot analysis (Supplementary Figure S4). Transfected BV2 cells were pretreated with OEA (50 μM) for 2 h before exposure to LPS for another 24 h. The data showed that stimulating the cells with LPS decreased the expression of Arg-1 and YM-1 compared with the negative control (Figure 6C,D). However, OEA treatment increased LPS-induced Arg-1 and YM-1 production in the negative control (Figure 6C,D), but the PPARα siRNA obviously antagonized the increased effect of OEA on Arg-1 and YM-1 protein expression (Figure 6C,D). These findings indicated that the increased Arg-1 and YM-1 expression induced by OEA was PPARα-dependent. Taken together, our results further demonstrated that OEA switched microglia from an M1-like phenotype to an M2-like phenotype through a PPARα-dependent pathway.

### 2.7. OEA Shifts Ischemic Neuron-Induced Microglia toward the M2 Polarization State through the PPARα Pathway

**Figure 7:**
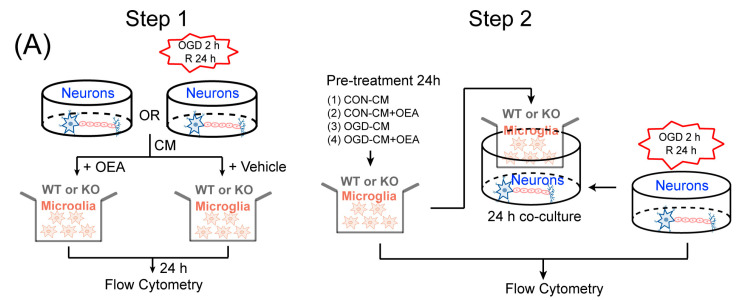

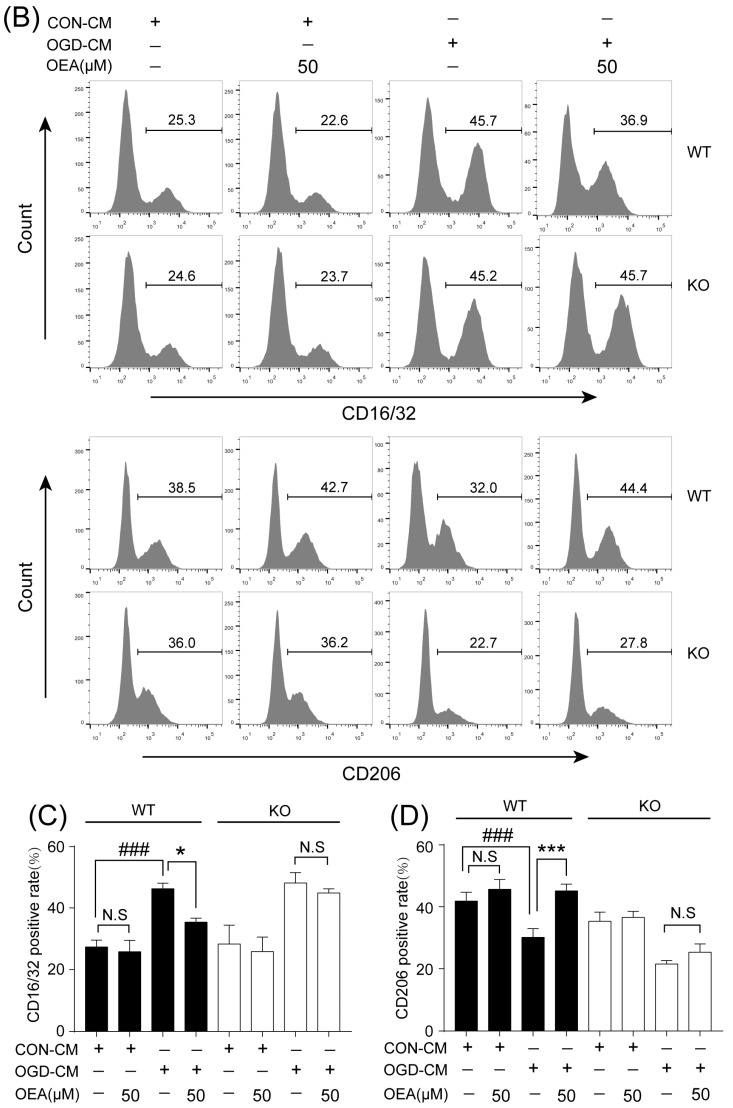
OEA shifts ischemic neuron-induced primary microglia polarization toward the M2 phenotype through PPARα. (**A**) In vitro experimental design. (**B**–**D**) Representative FACS panels and quantification showing that OEA promoted the M1-M2 switch in WT microglia, while it had no impact on the M1-M2 switch in KO microglia. The data are presented as the mean ± SEM. *n* = 5. Each experiment was repeated three times. ^###^ *p* < 0.001 vs. CON-neuron medium supernatant (CM) group. * *p* < 0.05, *** *p* < 0.001 vs. oxygen-glucose deprivation (OGD)-CM group; N.S = No Significance.

We utilized in vitro primary microglia or neuron cultures to extend our in vivo observations and further explore the cell-specific mechanisms of OEA-afforded neuroprotection. A previous study has indicated that ischemic neurons promote the transformation of microglia to the M1 phenotype [10,16]. In this study, we investigated whether OEA can transform ischemic induced-microglia polarization from M1 to M2 and whether the regulation effect of OEA on microglia polarization can be blocked through the knockout of microglial PPARα signaling (Figure 7A, Step 1). We exposed the primary neurons from WT mice cultures to 2 h of oxygen-glucose deprivation (OGD) and reperfusion for 24 h and then collected the neuron medium supernatant (CM) from healthy control (CON) neurons (CON-CM) or from OGD neurons (OGD-CM). Primary microglia from WT or PPARα-KO mice were treated with CON-CM or OGD-CM for another 24 h in the presence or absence of OEA. The microglial phenotype was examined using flow cytometry to detect the percentage of M1-like markers using CD16/32 and the M2-like marker using CD206. First, we found there was no difference in the levels of CD16/32 and CD 206 between the OEA+CON-CM group and the CON-CM group (Figure 7B–D), suggesting that there is no direct regulation by OEA on microglia polarization without ischemic stimulation. However, the data showed that OGD-CM increased the expression of CD16/32 and reduced the expression of CD206 in WT microglia (Figure 7B–D), suggesting that ischemic neurons shifted microglia toward the M1-like phenotype, which is consistent with a previous study [10]. However, OEA treatment significantly decreased the level of CD16/32 and markedly increased the level of CD206 in WT microglia (Figure 7B–D), indicating that the addition of OEA shifted microglia phenotype from an M1-like phenotype toward the M2-like phenotype upon stimulation with OGD-CM. However, these effects of OEA were not observed in PPARα-KO microglia (Figure 7B–D). Therefore, our data further confirmed that ischemic neurons induced microglia toward M1 polarization. However, OEA keeps ischemia-induced microglia in the M2 state through PPARα.

### 2.8. OEA Exerts an Indirect Neuroprotective Role in OGD-Induced Neurons by Enhancing Microglia M2 Polarization through the PPARα Pathway

**Figure 8:**
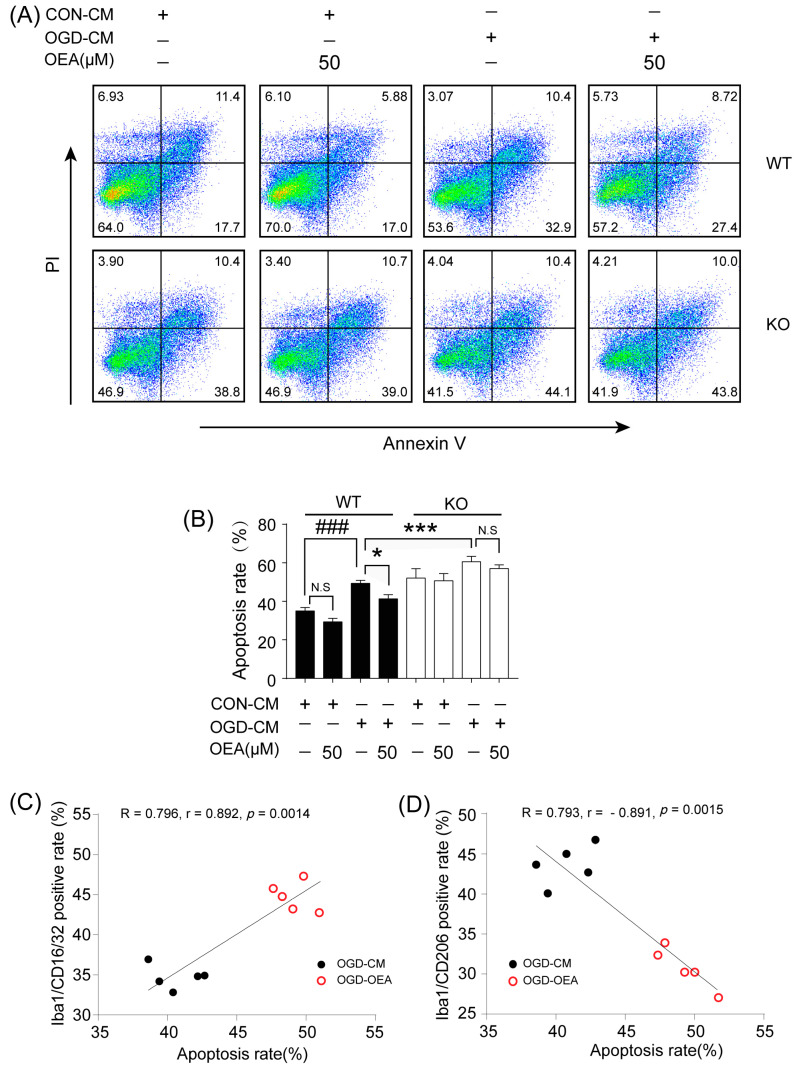
OEA treatment protects against OGD-induced neuronal apoptosis through PPARα. (**A**) Neuronal apoptosis was assessed with flow cytometry using annexin V-FITC staining. (**B**) The relative apoptosis ratio was analyzed. (**C**) Pearson correlation between Iba1/CD16/CD32 positive cells and neuronal apoptosis rate. (**D**) Pearson correlation between Iba1/CD206 positive cells and neuronal apoptosis rate. The data are presented as the mean ± SEM. *n* = 5. Each experiment was repeated 3 times. ^###^ *p* < 0.001 vs. CON-CM group. * *p* < 0.05, *** *p* < 0.001 vs. OGD-CM group; N.S = No Significance.

Next, we investigated whether OEA enhances neuronal survival by promoting the microglia M2-like phenotype in vitro (Figure 7A, Step 2). The primary microglia from WT or PPARα-KO mice seeded in culture inserts were treated with CON-CM or OGD-CM in the presence or absence of OEA for 24 h. These pretreated microglia were then added to the neuron cultures that had been subjected to 2 h OGD and reperfusion 24 h (Figure 7A, Step 2). Flow cytometry was used to quantify neuronal death at 24 h after coculture. Compared to the CON-CM group, the OGD-CM-treated M1-like WT microglia significantly enhanced neuronal death (Figure 8B). In contrast, the addition of OEA to OGD-CM-treated WT microglia (shifted to M2) dramatically decreased the death of OGD-induced WT neurons (Figure 8B). Additionally, we found that the neuroprotective effect of OEA on OGD-induced neurons disappeared in OGD-CM-treated PPARα-KO microglia, suggesting that OEA exerts a protective effect on neuronal damage through the PPARα pathway. Moreover, the data showed that the apoptosis rate for the neurons was increased with the OGD-CM-treated PPARα-KO microglia compared with the OGD-CM-treated WT microglia (Figure 8B), suggesting that PPARα deletion enhances OGD-induced neuronal death. Additionally, we have examined the effect of both neuron and microglia (N/G) co-cultures being exposed together to OGD and treated with OEA. Mouse N/G co-cultures were subjected to 2 h OGD and then treated with 50 μM OEA or vehicle (PBS). Neuronal survival was measured 24 h later using flow cytometry. We found that the apoptosis rate of neurons dramatically increased after OGD-treated in WT N/G co-cultures (Supplementary Figure S5A–C). However, OEA treatment markedly decreased the apoptosis rate of neurons in OGD-induced WT N/G co-cultures (Supplementary Figure S5A–C). Furthermore, we found that OEA has no neuroprotective effect on neurons in OGD-induced PPARα-KO N/G co-cultures, suggesting that OEA still plays a protective role in neurons through the PPARα pathway in N/G co-cultures. These results further confirm the positive role of PPARα signaling in neuroprotection after ischemia. To further determine whether OEA exerts its neuroprotective effects on OGD-induced neurons directly, we used two separate groups of microglia, CON-CM or CON-CM+OEA, which were pretreated and added to OGD-induced neuron coculture. Interestingly, we found that CON-CM+OEA treatment decreased the neuronal apoptosis rate, but there was no significant change compared with the CON-CM group (Figure 8B). Meanwhile, there was also no difference in the microglia polarization state between the CON-CM and CON-CM+OEA groups. Therefore, these data indicated that the neuroprotective effect of OEA was related to its promotion of microglia M2 polarization rather than its direct effect on neurons. Additionally, our results revealed that M1 microglia (OGD-CM group) positively correlated with the apoptosis rate of neurons (Figure 8C). However, the number of M2 microglia (OGD-OEA group) showed a significantly negative correlation with the apoptosis rate of the neurons (Figure 8D). These results indicated that OEA is capable of modulating microglial polarization toward the anti-inflammatory M2 phenotype and further demonstrates that the PPARα-mediated M2 microglia phenotype plays an essential role in neuron survival. Thus, we found that the activation of microglia PPARα signaling by OEA shifts their polarization toward the M2 state and further protects ischemic-induced neighboring neuron injury through the M2 phenotype.

## 3. Discussion

In the present study, we performed a set of in vivo and in vitro experiments to show that OEA markedly reduced neuronal damage after cerebral stroke. Additionally, OEA switches the microglia phenotype from an M1-like “pro-inflammatory” phenotype to an M2-like “anti-inflammatory” phenotype through the PPARα pathway. Furthermore, our data also indicated that the OEA-afforded neuroprotection relied on its modification of microglia M2 polarization through PPARα signaling.

Microglia are innate immune cells in the CNS. Distinct microglial polarization states are known to exert different effects on stroke pathology and brain repair [17]. M1-like microglia accelerate neuronal death and aggravate inflammation by generating a number of pro-inflammatory cytokines. However, M2-like microglia exert vital roles in repair and plasticity in the ischemic brain. Therefore, finding new compounds directed at shifting microglia from the M1 phenotype to the M2 phenotype may be a new strategy for the treatment of ischemic stroke [10]. With its anti-apoptotic, anti-oxidation and anti-inflammatory properties, OEA has been proven to be an effective neuroprotective agent in several CNS diseases [18]. Additionally, our previous study showed that OEA exerts a neuroprotective effect in both the acute and delayed stages of ischemic stroke [5,6,7,8,15]. Furthermore, we also found OEA inhibits inflammatory effects in LPS-induced THP-1 cells by enhancing PPARα signaling [19]. The positive effects of OEA on ischemic stroke have been confirmed in animal models. However, the exact mechanisms leading to this neuroprotection are still not fully understood, especially with regard to the microglia polarization status in cerebral ischemic injury. 

This study was conducted to analyze whether and how OEA induces microglia polarization after ischemic stroke. Hu et al. revealed that both the M1-like and M2-like markers in microglia/macrophages significantly increased from day 3 onward after stroke onset [11]. In the present study, brain samples 3 days after ischemia were chosen to detect the role of OEA in microglia/macrophage polarization. We found that the levels of M1-like markers (IL-1β, IL-6, TNF-α and CD16/32) and M2-like markers (Arg1, YM1 and CD206) were gradually increased 3 days after MCAO, which agrees with previous reports [11]. However, OEA treatment significantly inhibited the upgrading of M1 cytokines but further enhanced M2 marker expression. Therefore, our in vivo results indicated that OEA shifts microglia polarization toward the neuroprotective and beneficial M2 phenotype in the ischemic brain. To further confirm the protective effects of OEA were caused by microglia after MCAO, we performed a series of experiments using microglial BV2 cells and primary microglia in vitro. The data showed that OEA inhibited LPS-induced M1 polarization and facilitated IL-4-induced M2 polarization in BV2 microglia, suggesting that OEA can directly modulate the microglia polarization state. Meanwhile, we also found OEA reversed the LPS-induced decline of M2 markers in BV2 cells, further suggesting OEA could switch microglia polarization from the M1 to M2 phenotype. Ischemia-induced neurons primed primary microglia toward the M1 phenotype [16]. Our in vitro results also indicated that ischemia-induced WT neurons promote the polarization of WT microglia toward M1. However, OEA treatment shifted the WT microglia phenotype from the M1 phenotype toward the M2 phenotype. These results indicated that OEA could put ischemic neuron-induced primary microglia in the M2 polarization state. Interestingly, we found that OEA has no effect on the polarization of normal WT microglia when treated by CM from non-OGD-induced neurons. These data suggested that OEA can only regulate microglial polarization in the pathological state rather than typical physiological conditions. Therefore, our results suggested that OEA promoted microglia polarization to the M2 phenotype after ischemic stroke.

Previous studies have confirmed that M2 microglia promote ischemia-induced neuronal survival and enhance axon growth/sprouting in cultured neurons [16]. Consistent with previous reports, our study also indicates that increases in M2 polarization are significantly correlated with neuronal survival in both in vitro and in vivo cerebral ischemic models. Maintaining the microglia M2 phenotype could benefit cerebral injury in multiple ways, such as enhancing phagocytic activity and inhibiting the generation of inflammatory mediators [17]. Therefore, enhancing the recruitment of M2 microglia into the infarct border to restrict brain damage may be a new avenue of treatment using OEA in ischemic stroke. To confirm OEA exerts neuroprotective effects after cerebral ischemia by promoting the microglia M2 phenotype, we further used a primary microglia/neuron coculture system to elucidate the effect of OEA-induced microglial M2 phenotype on OGD-caused neuron injury. M1-polarized microglia induced by OGD+CM, M2-polarized microglia induced by OGD+CM+OEA, or nonpolarized microglia treated with CON+CM were applied to OGD-induced neuron cocultures. Consistent with previous research, our data showed that M1 microglia aggravated the post-OGD death of primary neurons. However, post-OGD primary neurons treated with the OEA-induced M2 microglia had a higher survival rate compared with those treated with M1 microglia only. Interestingly, OEA had no obvious effect on neuronal injury when neurons were cocultured with nonpolarized microglia. These results indicate that the protective effect of OEA on neurons was mediated through microglia M2 polarization induced by itself rather than directly on neurons. Therefore, our in vitro results further confirmed that maintaining microglia M2 status indeed exerts a neuroprotective role after ischemic injury. Taken together, we found that OEA’s neuroprotection against ischemic neuron injury is attributed to its promotion of the microglia M1–M2 switch rather than its direct effect on neurons. 

Microglia are involved in inducing the recruitment of immune cells and inflammatory cytokines after ischemic stroke. The microglia/macrophage M2-to-M1 shift during chronic inflammation after stroke expands neuronal damage and reduces neuronal self-restorative abilities [17]. There is also a similar M2-to-M1 switch in models of traumatic brain injury (TBI) [20] and spinal cord injury (SCI) [21], suggesting that the microglia/macrophage phenotypic shifts may be a common pathologic process in multiple types of CNS diseases. However, the lack of necessary endogenous signals/targets for M2 induction leads to worsened outcomes after cerebral ischemia. Therefore, finding new endogenous signals/targets to regulate the M2-to-M1 switch with microglia-directed therapies may present a benefit to not only victims of stroke but also other neurologic diseases. The PPARα receptor is ubiquitously expressed in microglial cells [22]. Our prior report showed that PPARα deficiency promotes an inflammation response [19]. Here, our in vivo data showed that OEA could not shift microglia/macrophage polarization toward the M2 phenotype in PPARα-KO mice after ischemic stroke. Meanwhile, OEA enhanced the activation of PPARα and further increased PPARα binding activity with M2 marker promoters, which will lead to the promotion of transcriptional activity of M2 genes. However, these changes were not observed in PPARα-KO mice. These results indicated that OEA promotes microglia/macrophage polarization toward the M2 phenotype through the activation of PPARα and M2 genes, which are the direct PPARα target genes. Our in vitro studies also showed that PPARα deficiency obviously antagonized the promoting effect of OEA on the microglia M2 transition. Thus, these results strongly demonstrated OEA switched microglia polarization from M1 to M2 through a PPARα-dependent pathway, and PPARα may be an endogenous target in the regulation of microglia phenotypes after stroke. Therefore, in addition to regulating blood lipids [12], inhibiting inflammation [13] and promoting learning and memory [14], a new biological function of PPARα may be the regulation of microglial polarization. However, how PPARα participates in this M2-M1 phenotype switch in chronic inflammation after a stroke still needs further study. The neuroprotective effects of PPARα have been confirmed in several disease models, such as stroke, traumatic brain injury, Parkinson’s disease, Alzheimer’s disease and diabetic peripheral neuropathy [23,24,25,26]. Previous studies have reported that PPARα’s neuroprotective effects in ischemic stroke are related to the inhibition of ischemia-induced oxidative stress and inflammation [27]. Our previous studies have demonstrated OEA protects mice from focal cerebral ischemic injury and attenuates neuronal apoptosis through the PPARα pathway [5,7]. These findings indicated that PPARα is a potential therapeutic target after ischemic stroke. In this study, we found that the neuroprotective effects of OEA disappeared in injured neurons cocultured with microglia with the deletion of the PPARα signal, indicating that OEA exerts a protective effect on neuronal damage through microglial PPARα signaling. Additionally, our data also showed that PPARα deletion enhances OGD-induced neuronal death, which further confirms the beneficial role of PPARα signaling in neuroprotection after ischemia. Therefore, we report for the first time that PPARα is not only involved in microglial polarization but also in the neuroprotective effect of the beneficial microglia M2 phenotype. Taken together, we found that the activation of microglia PPARα signaling by OEA shifts their polarization toward the M2 state and further protects ischemic-induced neighboring neurons injury through the M2 phenotype. However, we here mainly focused on the protective effect of OEA on acute stroke, but the effect of OEA on other types of strokes (such as hemorrhagic stroke and lacunar infarcts) is still unclear [28]. Therefore, additional research in the future is necessary to evaluate the neuroprotective effect of OEA on hemorrhagic stroke and lacunar infarcts.

## 4. Methods and Methods

### 4.1. Animals

All animal procedures were in strict accordance with the National Institutes of Health’s Guidelines for the Care and Use of Laboratory Animals. All the experimental protocols were approved by the Animal Care and Use Committee of Xiamen University following the Guide for the Care and Use of Laboratory Animals (8th edition, 2011) [29]. PPARα-knock-out (KO) mice (129S4/SvJae-Pparatm1Gonz/J) and the corresponding control 129S4/SvJae (WT) mice were obtained from the Jackson Laboratory (Bar Harbor, ME, USA). All mice were housed under a 12/12-h dark/light cycle and specific pathogen-free (SPF) conditions. Additionally, they were bred at the Animal Center of Xiamen University. The animals fasted for 12 h before the MCAO procedure was performed. The animals were randomly divided into sham, MCAO and MCAO+OEA groups, and 5 mice from each group were euthanized at each time point, and the brain tissue was collected for various assessments, which were performed by 2 investigators in a blinded manner.

### 4.2. The Focal Cerebral Ischemia Model and Drug Administration

Focal cerebral ischemia was induced by the intraluminal occlusion of the left middle cerebral artery (MCA) for 90 min, as previously described [7]. Briefly, male WT and PPARα-KO mice at 7–9 weeks old were anesthetized with an isoflurane-based mixture delivered by a mask. Then, a 6-0 nylon monofilament suture with rounded tips was introduced into the right internal carotid artery (ICA) through the external carotid stump and advanced approximately 10 mm past the ECA/ICA bifurcation to occlude the origin of the middle cerebral artery (MCA) at the junction of the circle of Willis. Sham-operated mice received an identical surgery, except that the intraluminal filament was not inserted. Throughout the procedure, body temperature was maintained at 37 ± 0.5 °C. Mice were excluded if a hemorrhage was found in the brain slices or at the base of the circle of Willis during postmortem examination.

OEA (O0383) was purchased from Sigma (St. Louis, MO, USA). Our previous study indicated that OEA at a dose of 40 mg/kg had the greatest effect against ischemic stroke. In the present study, mice received i.p. injections of OEA (dissolved in 10:90 Tween-20: saline, 40 mg/kg) at the time of reperfusion. Tween-20: saline (10%, *v*/*v*) was used as a vehicle control.

### 4.3. Cell Culture and Drug Treatments

Dulbecco’s modified Eagle’s medium (DMEM), Neurobasal^TM^-A medium, fetal bovine serum (FBS), penicillin and streptomycin were purchased from Gibco BRL (Carlsbad, CA, USA). LPS (L4391) and IL-4 (SRP3211) were purchased from Sigma (St. Louis, MO, USA). 

BV2 cell culture. The murine microglia cell line BV-2 was kindly provided by Dr. Xiaofen Zheng (School of Medicine, Xiamen University, Xiamen, China) and was maintained in DMEM with 10% FBS, 100 U/mL penicillin, and 100 g/mL streptomycin at 37 °C in a 5% CO_2_ atmosphere. The BV-2 cells were treated with OEA at the indicated concentrations and incubations at a cell density of 2 × 10^6^ cells per 35-mm well. OEA was dissolved in dimethyl sulfoxide (DMSO), and the final DMSO concentration was less than 0.05%. Before LPS (1 μg/mL) or IL-4 (20 ng/mL) stimulation, BV-2 cells were pretreated for 2 h with 3 different concentrations (10, 30 and 50 μM) of OEA in 0.05% DMSO, and the cells were harvested after 12 h for RT-PCR testing. A siRNA assay was performed to silence PPARα in BV2 cells. Briefly, 0.75 × 10^6^ cells were diluted in fresh medium and transferred to 12-well plates at 24 h before transfection. For transfection, all siRNAs (PPARα or the negative control) were resuspended to a final concentration of 40 nM. After 24 h of incubation, the transfected cells were pretreated with 50 μM OEA for 2 h before stimulation with 1 μg/mL LPS. The total mRNA or protein contents were isolated, and the supernatants were collected after 12 h or 24 h stimulation. A transfection efficiency of 65–75% of cells was found for all experiments.

Primary neuron cultures and conditioned media collection. Primary cortical neuronal cultures were prepared from 17-d-old mixed-sex WT and PPARα-KO mice embryos, as previously described [30]. To model ischemia in vitro, neuron cultures were exposed to transient OGD for 2 h. Control cultures were incubated for the same period of time at 37°C in humidified 95% air and 5% CO_2_. The conditioned media (CM) were collected from OGD neurons (OGD-CM) or healthy control neurons (CON-CM) 24 h later and concentrated using centrifugal filters (Millipore, Burlington, MA, USA).

Primary microglia cultures and treatments. Primary microglia cultures were prepared from the whole brains of 1-d-old WT and PPARα-KO mice pups, as previously described [11]. The microglia were treated with OGD-CM in the presence or absence of 50 µM OEA and CON-CM for 24 h. The cells were then collected for flow cytometry assessment.

Neuron–microglia cocultures. Primary microglia (1 × 10^5^/well) were seeded in culture inserts and treated with OGD-CM or CON-CM in the presence or absence of OEA for 24 h. These pretreated microglia were then added to 11-d-old neurons that had been subjected to 2 h OGD or sham conditions in the presence of 50 µM OEA. Neuronal survival was analyzed 24 h after coculture using flow cytometry.

### 4.4. Immunohistochemistry and Cell Counting

Immunohistochemistry analysis was performed on 30-μm free-floating sections [6]. The sections were incubated in PBS containing 0.5% Triton X-100 and 10% normal goat serum for 1 h at room temperature and then with mice monoclonal Iba1 (1:500; Abcam, Cambridge, UK), rabbit polyclonal CD16/32 (1:500; Abcam, Cambridge, UK), rabbit polyclonal CD206 (1:500; Abcam, Cambridge, UK) and mice monoclonal NeuN (1:500; Abcam, Cambridge, UK) at 4 °C overnight. After several PBS rinses, sections were incubated with Alexa Fluor 488 donkey anti-mice IgG (1:500; Invitrogen, Carlsbad, CA, USA) or Alexa Fluor 596 donkey anti-rabbit IgG (1:500; Invitrogen, Carlsbad, CA, USA). An experimenter (Z.H.J) coded all slides from the experiments before quantitative analysis. The number of Iba1/CD16/32-, Iba1/CD206- or NeuN-positive cells was analyzed using fluorescence confocal microscopy (EX61, Olympus, Tokyo, Japan) by another experimenter (X.L.X) blinded to the study code. The means were calculated from 3 randomly selected microscopic areas in the cortex and striatum of each section, and 5 consecutive sections were analyzed for each brain. The data are expressed as the mean numbers of cells per square millimeter.

### 4.5. Real-Time PCR

The total RNA was isolated from brain tissues or BV2 cells using the RNeasy Mini Kit (Qiagen, Hilden, Germany) according to the manufacturer’s instructions. Real-time PCR was performed with the Fast Start Universal SYBR Green Master Mix (Roche Applied Science, Penzberg, Germany) on an ABI PRISM 7500 Sequence Detection System using corresponding primers (Table 1). The Ct values for the gene expression of TNF-α, IL-6, IL-1β, Arg1, CD206 and YM1 were normalized to the corresponding values for GAPDH gene expression.

### 4.6. Western Blot

The total protein content was extracted from the penumbral area of the ischemic hemispheres and BV2 cells. Brain tissue in the ischemic hemisphere was homogenized with lysis buffer (50 nM/L NaCl, 1 mM/L EDTA, 1% Triton X-100, 0.5% SDS, 0.5% sodium deoxycholate and 20 mM/L Tris HCl; pH 7.5) and centrifuged at 15,000× *g* for 20 min. Protein samples (50 μg per lane) were run on a polyacrylamide gel, transferred to a PVDF membrane (Millipore, Billeria), and blocked with a 5% milk solution (nonfat dry milk in PBST) for 2 h. Then, the membranes were incubated with the primary antibodies against TNF-α, IL-1β, IL-6, Arg1 and YM1 and β-actin (1:1000, all from Cell Signaling Technology, Danvers, MA, USA) overnight at 4 °C. After washing with TBST 3 times, and the membranes were then incubated with the corresponding conjugated anti-rabbit IgG (1:10,000; Cell Signaling Technology) at room temperature for 2 h. Immunoreactivity was detected by an enhanced chemiluminescence (ECL) kit (Millipore, Billeria), and the relative densities of the protein bands were scanned using a LAS 4000 Fujifilm imaging system (Fujifilm, Tokyo, Japan) and analyzed by densitometric evaluation using Quantity-One software Version 4.6.6 (Bio-Rad Hercules, CA, USA).

### 4.7. PPARα Transcriptional Activity Assay

The PPARα activity was assessed in nuclei obtained from brains 3 d after MCAO or sham procedure using a PPARα Transcription Factor Assay kit (Cayman Chemical Company, Ann Arbor, MI, USA). Briefly, nuclear extracts were incubated in a PPRE probe-coated multiwell plate, and PPAR bound to the PPRE probe was detected using a specific antibody against the α isoform. A horseradish peroxidase-labeled secondary antibody was used and detected by spectrophotometry.

### 4.8. Chromatin Immunoprecipitation

Chromatin immunoprecipitation (ChIP) assays were performed using the EZ-ChIP Kit (Sigma-Aldrich MI, IT, USA) according to the manufacturer’s instructions. The genomic DNA was sonicated, immunoprecipitated with an anti-PPARα antibody certified for ChIP analysis (Thermo; 3B6/PPAR) and subsequently released from histones by following a previously-described procedure. Then, the DNA sequences were amplified by real-time PCR using the following primers against the PPARα site in the Arg1 promoter (Forward: GCATCCAAGACTTAAGCCCAGC; Reverse: CTTGGTGCTGGCCCACAAAT) and YM-1 (Forward: TCAGCCATGCATTCAAACTTTGGA; Reverse: TCTTTGCAAGACACACACACAGAC). PCR conditions were as described previously.

### 4.9. Flow Cytometry Analysis

The primary microglia phenotype induced with OGD-CM or CON-CM in the presence or absence of OEA was stained with CD16/32 (Invitrogen, Waltham, MA, USA) and CD206 (Proteintech, Rosemont, IL, USA) fluorescently labeled antibodies. The primary microglia from each group were collected and resuspended in PBS at 1 × 10^6^ cells/mL. After centrifugation at 300× *g* for 5 min, the microglia were stained with the CD16/CD32 antibody (0.25 μg/test) and CD206 antibody (0.2 μg/test), fixed with 4% PFA, and blocked with 3% BSA at room temperature for 30 min. The data were assessed by flow cytometry (Gallios; Beckman, Indianapolis, IN, USA) and analyzed with the FlowJo X software. The OGD-induced neuron apoptosis in neuron–microglia cocultures was detected using an Annexin V-FITC/PI apoptosis detection kit (Yeasen Corporation, Shanghai, China). Neurons in the lower layer of the transwell chamber from each group were collected and resuspended in PBS at 1 × 10^5^ cells/mL. After centrifugation at 300× *g* for 5 min, 100 μL of binding buffer was added to resuspend the neurons. Then, 5 μL of Annexin V-FITC and 5 μL of PI Staining Solution were added to the neurons. After incubation at room temperature for 10 min in the dark, 400 μL of binding buffer was added to each sample. Flow cytometry (Gallios; Beckman, Indianapolis, IN, USA) was used to detect the apoptosis of neurons, and the data were analyzed using FlowJo X software.

### 4.10. Statistical Analysis

All values are expressed as the mean ± SEM. The significant differences among means of multiple groups were assessed by 1 or 2-way ANOVA followed by Tukey’s post hoc test (Prism 7 for Windows, GraphPad Software Inc., Boston, MA, USA). The Pearson product linear regression analysis was used to correlate the neuroprotective effects and microglia M2 phenotype. Significance was determined to be *p* < 0.05.

## 5. Conclusions

In summary, we find the novel effects of OEA in enhancing microglia M2 polarization to protect neighboring neurons by activating the PPARα signal, which is a new mechanism of OEA against cerebral ischemic injury. Therefore, OEA might be a promising therapeutic drug for stroke and targeting PPARα-mediated M2 microglia may represent a new strategy to treat ischemic stroke.

## Figures and Tables

**Table 1:** Primer sequences for quantitative real-time PCR assay.

Gene	Primer Sequence
Arg1	5′-TCACCTGAGCTTTGATGTCG-3′
	5′-CTGAAAGGAGCCCTGTCTTG-3′
Ym1	5′-CAGGGTAATGAGTGGGTTGG-3′
	5′-CACGGCACCTCCTAAATTGT-3′
CD206	5′-CAAGGAAGGTTGGCATTTGT-3′
	5′-CCTTTCAGTCCTTTGCAAGC-3′
IL-1β	5‘-CTCACAAGCAGAGCACAAGC-3′
	5′-CAGTCCAGCCCATACTTTAGG-3′
IL-6	5′-CGGAGAGGAGACTTCACAGAG-3′
	5′-CATTTCCACGATTTCCCAGA-3′
TNFα	5′-TATGGCTCAGGGTCCAACTC-3′
	5′-GGAAAGCCCATTTGAGTCCT-3′
GAPDH	5′-AAGATGGTGAAGGTCGGTG-3′
	5′-GTTGATGGCAACAATGTCCAC-3′

## Data Availability

The data is contained within the article and supplementary materials.

## Supplementary Materials

The following supporting information can be downloaded at: https://www.mdpi.com/article/10.3390/ph16040621/s1, Figure S1: OEA treatment inhibits microglia M1 polarization in the peri-infarct of the cortex and striatum in WT but not KO mice at 3 days after MCAO.; Figure S2: OEA treatment promotes microglia M2 polarization in the peri-infarct of the cortex and striatum in WT but not KO mice at 3 days after MCAO; Figure S3: Effects of OEA on BV2 cells viability. BV2 cells were cultured with different concentrations of OEA (10, 30, and 50 μM) for 24 h; Figure S4: BV2 cells were transiently transfected with PPARα siRNA for 24 h, and the protein expression of PPARα were subsequently measured by western blot; Figure S5: OEA treatment protects against neuronal apoptosis after OGD-treated in N/G co-cultures through PPARα.

## Abbreviations

OEA, oleoylethanolamide; PPARα, peroxisome proliferator-activated receptor α; WT, wild-type; KO, knock out; OGD, oxygen-glucose deprivation; SPF, specific pathogen-free; MCAO, middle cerebral artery occlusion; ICA, internal carotid artery; FBS, fetal bovine serum; DMSO, dimethyl sulfoxide; CM, conditioned media; ChIP, Chromatin immunoprecipitation; TBI, traumatic brain injury; SCI, spinal cord injury; MTT, 3-(4,5-dimethyl-2-thiazolyl)-2,5-diphenyl-2-H-tetrazolium bromide.
