# Supplementary material for: Oleoylethanolamide Protects against Acute Ischemic Stroke by Promoting PPARα-Mediated Microglia/Macrophage M2 Polarization
Source: Pharmaceuticals (Basel). 2023 Apr 20;16(4):621. doi: 10.3390/ph16040621 (PMC10146893; doi:10.3390/ph16040621)
Supplement: Supplementary file 1 [file pharmaceuticals-16-00621-s001.zip › pharmaceuticals-2255984-supplementary.pdf]

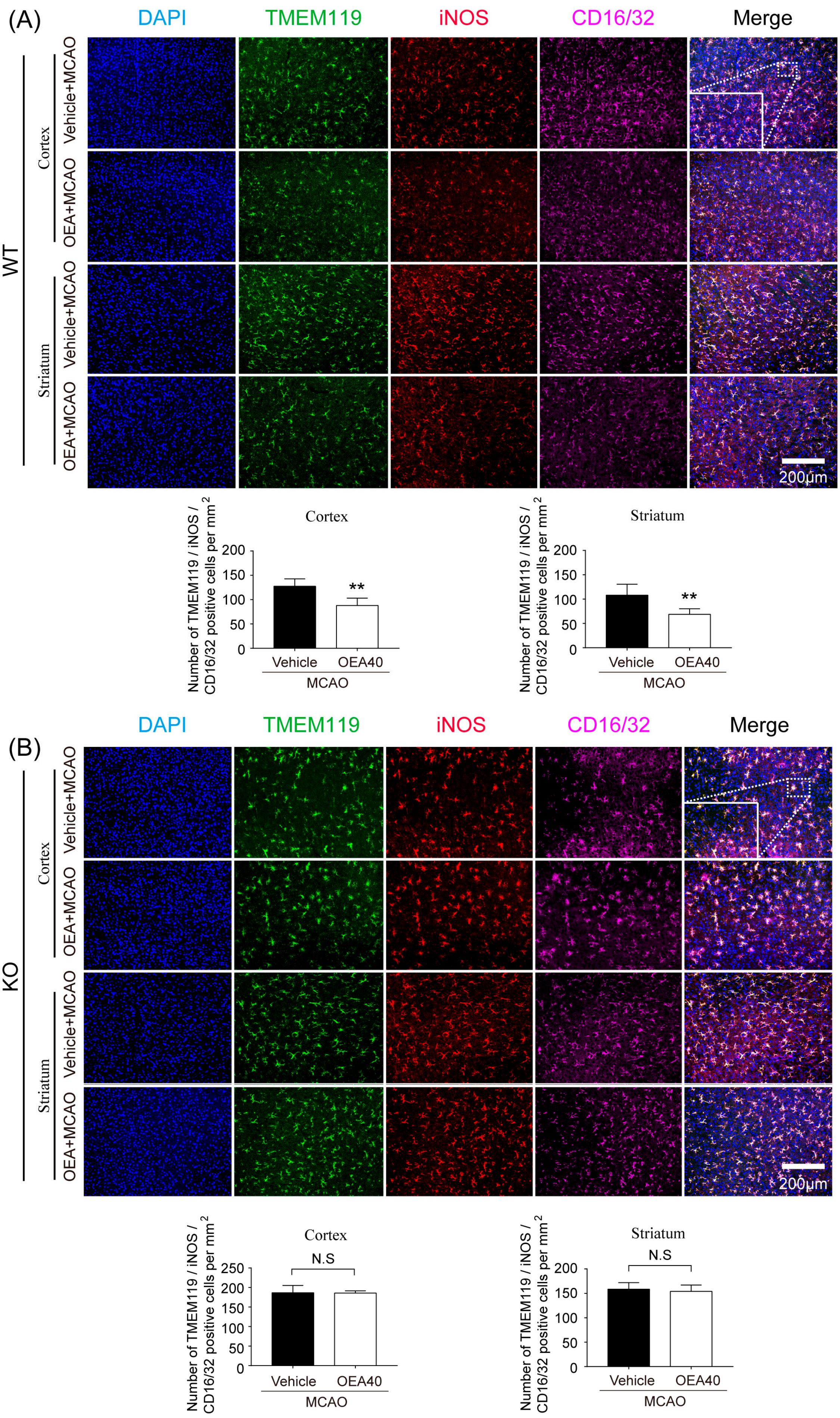
(A)
DAPI
TMEM119
iNOS
CD16/32
Merge
WT
Cortex
Striatum
Vehicle+MCAO
OEA+MCAO
200μm
Cortex
Striatum
Number of TMEM119 / iNOS / CD16/32 positive cells per mm2
200
150
100
50
0
**
Vehicle
OEA40
MCAO
(B)
KO
N.S
250

Figure S1. OEA treatment inhibits microglia M1 polarization in the peri-infarct of the cortex and striatum in WT but not KO mice at 3 days after MCAO. (A) Representative immunofluorescence images and quantification of TMEM119, iNOS and CD16/32 in the peri-infarction of WT mice on day 3 after MCAO. (B) Representative immunofluorescence images and quantification of TMEM119, iNOS and CD16/32 in the peri-infarction of KO mice on day 3 after MCAO. The data are the means ± SEM. n = 5 per group. $^{**}p < 0.01$ *vs* MCAO + vehicle group; N.S = No Significance. Scale Bar=100 μm.

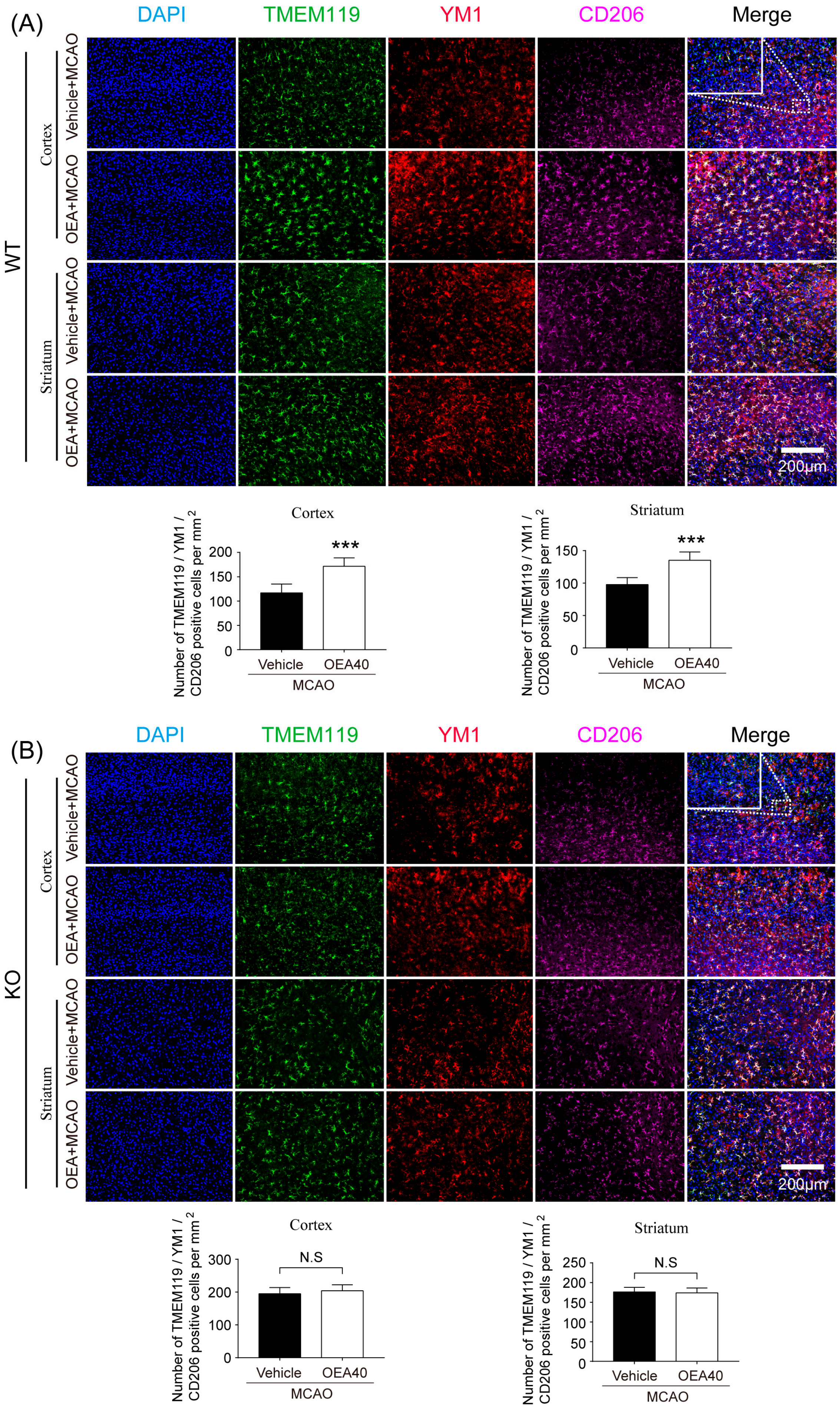
(A)
DAPI
TMEM119
YM1
CD206
Merge
WT
Cortex
Striatum
Vehicle+MCAO
OEA+MCAO
Vehicle+MCAO
OEA+MCAO
200μm
Cortex
Number of TMEM119 / YM1 / CD206 positive cells per mm2
200
150
100
50
0
***
Vehicle
OEA40
MCAO
Striatum
Number of TMEM119 / YM1 / CD206 positive cells per mm2
150
100
50
0
***
Vehicle
OEA40
MCAO
(B)
DAPI
TMEM119
YM1
CD206
Merge
KO
Cortex
Striatum
Vehicle+MCAO
OEA+MCAO
Vehicle+MCAO
OEA+MCAO
200μm
Cortex
Number of TMEM119 / YM1 / CD206 positive cells per mm2
300
200
100
0
N.S
Vehicle
OEA40
MCAO
Striatum
Number of TMEM119 / YM1 / CD206 positive cells per mm2
250
200
150
100
50
0
N.S
Vehicle
OEA40
MCAO

Figure S2. OEA treatment promotes microglia M2 polarization in the peri-infarct of the cortex and striatum in WT but not KO mice at 3 days after MCAO. (A) Representative immunofluorescence images and quantification of TMEM119, YM1 and CD206 in the peri-infarction of WT mice on day 3 after MCAO. (B) Representative immunofluorescence images and quantification of TMEM119, YM1 and CD206 in the peri-infarction of KO mice on day 3 after MCAO. The data are the means ± SEM. n = 5 per group. $^{***}p < 0.001$ *vs* MCAO + vehicle group; N.S = No Significance. Scale Bar=100 μm.

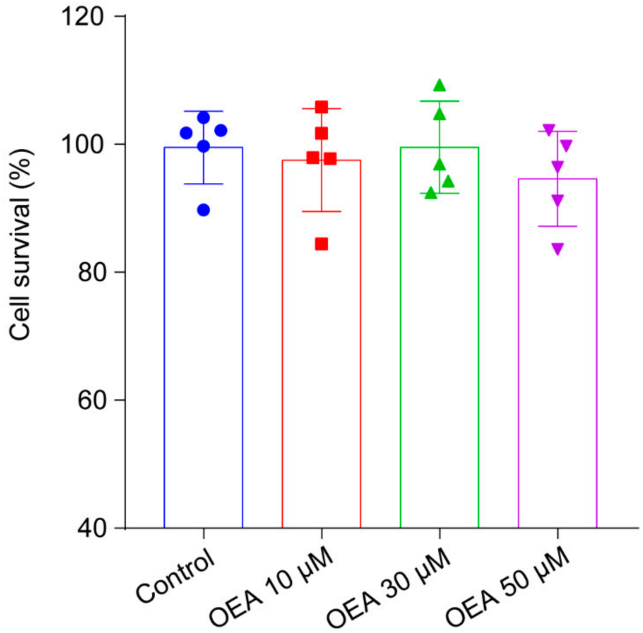

Figure S3. Effects of OEA on BV2 cells viability. BV2 cells were cultured with different concentrations of OEA (10, 30, and 50 μM) for 24 h. Cell viability was assessed using an MTT assay. The values are presented as means ± SEM of five independent experiments performed in duplicate (n = 5). $^{\#}p < 0.05$ *vs.* control group.

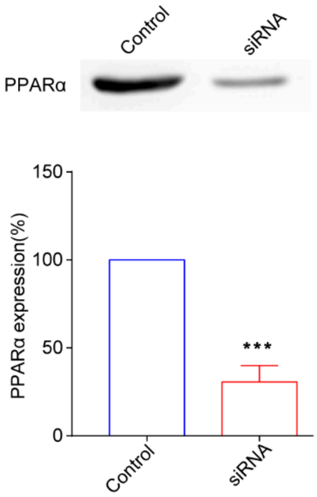

Figure S4. BV2 cells were transiently transfected with PPAR*α* siRNA for 24 h, and the protein expression of PPAR*α* were subsequently measured by western blot. The values are presented as means ± SEM of five independent experiments performed in duplicate (n = 5). $^{\#\#\#}p < 0.001$ *vs.* control group.

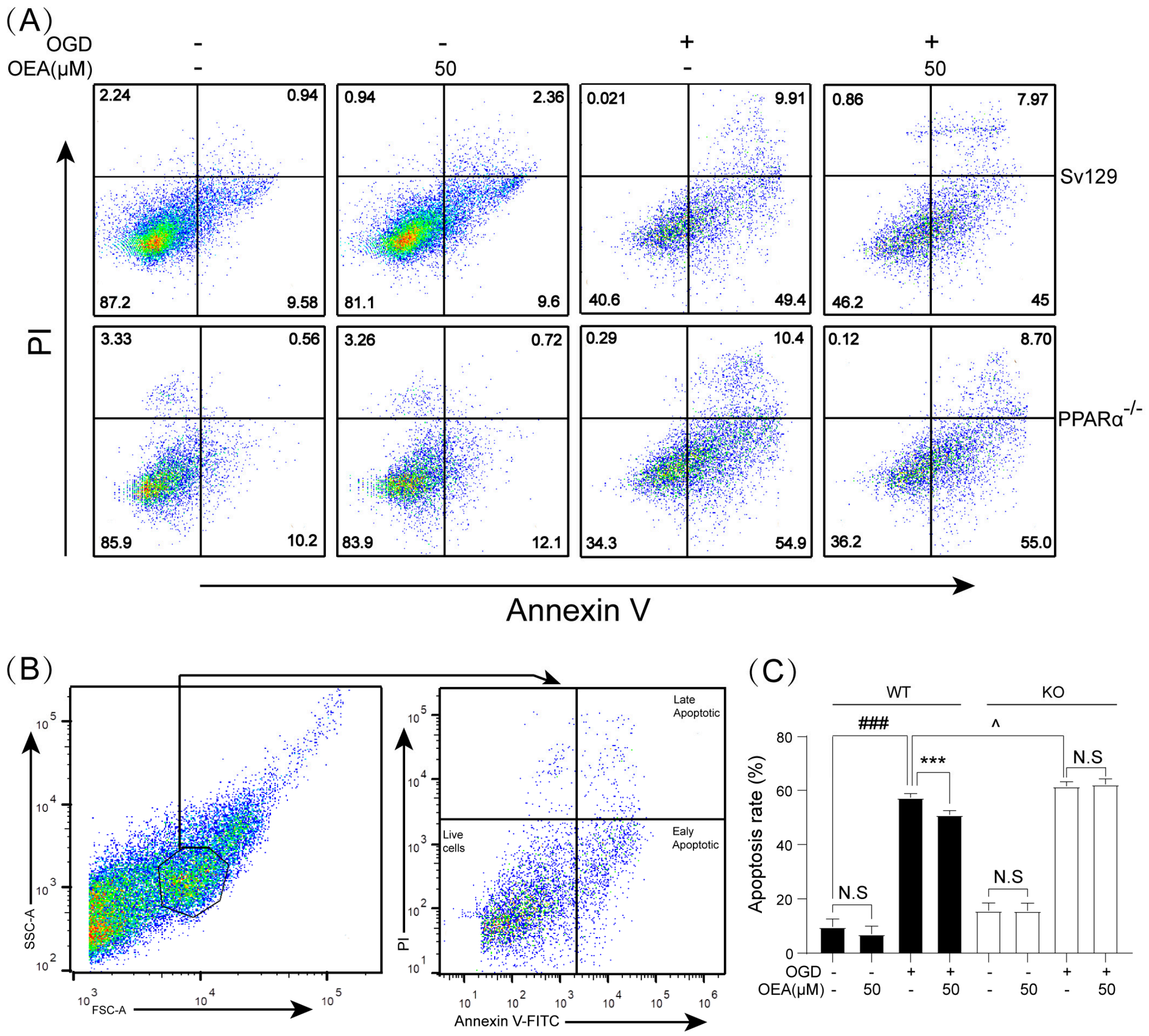

Figure S5. OEA treatment protects against neuronal apoptosis after OGD-treated in N/G co-cultures through PPAR*α*. (A) Neuronal apoptosis was assessed with flow cytometry using annexin V-FITC staining. (B and C) The relative apoptosis ratio was analyzed. The data are presented as the mean ± SEM. N = 5. Each experiment was repeated 3 times. $^{###}p < 0.001$ *vs*. Control group, $^{***}p < 0.001$ *vs*. OGD group, $^{\wedge}p < 0.05$ *vs*. WT OGD group
